# Polymorphism of the *CSN3* 3’UTR in Dairy Cows Causes Changes in bta-miR-708 Binding Ability and κ-Casein Expression

**DOI:** 10.3390/ani14233462

**Published:** 2024-11-29

**Authors:** Wenqing Li, Xiaoyang Wang, Pinhui Wu, Xiuyang Xu, Wei Liu, Guozhi Zhang, Liyang Zhang, Tong Fu, Tengyun Gao

**Affiliations:** 1 College of Life Science, Henan Agricultural University, Zhengzhou 450046, China; liwenqing605@163.com (W.L.); wangxiaoyang312@163.com (X.W.); phwu2022@163.com (P.W.); xxy15713631303@163.com (X.X.); liuv@henau.edu.cn (W.L.);; 2 College of Animal Science and Technology, Henan Agricultural University, Zhengzhou 450046, China; futong2004@126.com

**Keywords:** *CSN3*, 3’UTR, polymorphism, miRNA, haplotype

## Abstract

κ-casein (*CSN3*) polymorphisms are closely related to productive performance and dairy processing performance. In this study, *CSN3* 3’UTR polymorphisms in Chinese Holstein cows (n = 50) and the effects of these polymorphisms on the gene expression of four caseins, the protein expression of κ-casein, the binding of microRNAs (miRNAs), and cow production performance were investigated. There were two main haplotypes for the *CSN3* 3’UTR in Chinese Holstein cows: Haplotype 1 (0 mutations, n = 16) and Haplotype 10 (a combination of 7 mutations, n = 17). The gene and protein expression levels of *CSN3* in Haplotype 1 were significantly greater than those in Haplotype 10 (*p* < 0.05). The ability of bta-miR-708 to bind to the *CSN3* 3’UTR with Haplotype 10 was significantly greater than that with Haplotype 1 (*p* < 0.05), potentially explaining why the gene and protein expression of *CSN3* with Haplotype 10 were lower than those with Haplotype 1. Dairy herd improvement (DHI) data, including 305-d milk production, fat-corrected milk, fat (%), protein (%), and fat/protein ratio, were not significantly different between the two haplotypes (*p* > 0.05). Results suggest that the two major *CSN3* 3’UTR polymorphisms in Chinese Holstein cows cause significant changes in *CSN3* gene expression, CSN3 protein expression, and bta-miR-708 binding ability but no significant alterations in macroscopic DHI data. Therefore, *CSN3* 3’UTR polymorphisms should be investigated further.

## Simple Summary

κ-casein polymorphism is closely related to productive performance and dairy processing performance. In this study, the polymorphism of *CSN3* 3’untranslated regions (3’UTR) in Chinese Holstein cows and the effect of polymorphism were investigated. The *CSN3* 3’UTR polymorphism was characterized by two main haplotypes. There were significant differences in gene and protein expression of CSN3 between the two haplotypes. The binding abilities of bta-miR-708 to recombinant vectors with these two haplotypes were significantly different, potentially explaining why the gene and protein expressions of CSN3 with two haplotypes were different.

## 1. Introduction

Approximately 80% of the proteins in milk are encoded by four casein genes, which are tightly linked and form casein gene clusters on chromosome 6: *CSN1S1*, *CSN2*, *CSN1S2*, and *CSN3*, which encode αs1-casein, β-casein, αs2-casein, and κ-casein, respectively [1,2]. Bovine κ-casein is located on the surface of casein micelles and is a Ca^2+^-insensitive protein [3,4]. The amount of glycosylated κ-casein was shown to be associated with casein micelle size [5]. It was reported that κ-casein is also associated with milk coagulation properties and some cheese characteristics [6]. The *CSN3* gene was confirmed to be the most important gene for an individual cow’s cheese yield [7].

A variation in the coding region of casein genes alters the amino acid sequence and affects the functional properties of the protein [8]. Thus, milk protein variants in the coding sequences have been widely studied [9,10]. However, does a variation in the noncoding region also affect casein function or expression?

Some DNA variants in noncoding sequences, such as 5′untranslated regions (5’UTR), including promoters, 3′untranslated regions (3’UTR), and introns or intragenic regions, were shown to alter specific protein expression and, as a consequence, milk composition and cheesemaking [9,11]. Important mutations of *CSN1S1* in noncoding sequences have been reported: for example, a single point mutation results in A allele-specific exon skipping in bovine *CSN1S1* mRNA and reduces α_s1_-casein expression [12]; a 371 insertion in noncoding exon 19 of the bovine CSN1S1*G allele is associated with a lower proportion of αs1-casein in milk [13]; a polymorphism in the bovine *CSN1S1* promoter has significant effects on protein percentage [14]. For *CSN3*, Damiani et al. reported that a single-nucleotide polymorphism (SNP) in the second intron of *CSN3* was associated with several milk production traits [15]. These noncoding variants might affect splice sites, mRNA stability, or the transcription efficiency of mRNA, consequently leading to the loss of truncated protein products [9,16].

At present, there are few reports on important mutations in the noncoding region of bovine *CSN3* and their effects, especially in the 3’UTR. Important mutations are those that alter specific protein expression, which in turn affects milk protein composition and cheese production, or rather, mutations that are highly relevant to milk production and processing characteristics. To prevent adverse mutations from occurring, constant monitoring for milk protein mutations is needed. In this study, *CSN3* 3’UTR polymorphisms in 50 Chinese Holstein cows were detected, and the potential effects of *CSN3* 3’UTR polymorphisms were revealed. The results of this study can be used as a reference for breeding and genetic improvements in dairy cows.

## 2. Materials and Methods

### 2.1. Ethics Approval

The entire experiment was approved by the Animal Welfare and Research Ethics Committee of Henan Agricultural University (HNND2024031807, 18 March 2024). All efforts were made to minimize suffering by the animals.

### 2.2. Animals and Samples 

All 50 healthy Chinese Holstein cows were obtained from the Qi County Farm in Kaifeng, Henan Province, China. Two 15 mL milk samples were collected from each cow in March 2022. The milk samples were placed in an incubator with an ice bag and transported quickly to the laboratory. Each sample was centrifuged at 2000× *g* for 10 min at 4 °C to isolate the fat in the upper layer and the milk whey in the middle layer to obtain somatic cells. Somatic cells from one 15 mL sample were mixed with 1 mL of TRIzol, transferred to a 1.5 mL RNase-free centrifuge tube, and stored at −80 °C for subsequent RNA extraction; the somatic cells from the second 15 mL sample were stored at −20 °C for subsequent DNA extraction. The sampling method for the DHI data was as follows. Potassium dichromate (0.03 g) was added to the samples as a preservative. A total of 40 mL of milk was sampled per cow. After sampling, the sample was shaken, stored at 4 °C, and sent to Henan Dairy Herd Improvement Co., Ltd. (Zhengzhou, China) within 24 h.

### 2.3. RNA Extraction and cDNA Synthesis

RNA was isolated from each sample using a TRIzol reagent (Invitrogen, CA, USA) according to the manufacturer’s instructions. Total RNA extracts were employed to synthesize cDNA using a PrimeScript™ One Step RT‒PCR Kit (TaKaRa, Otsu, Japan).

### 2.4. Full-Length CSN3 3’UTR Amplification and Sequencing

The above cDNA was used as a template to amplify the full-length (207 bp) *CSN3* 3’UTR. The following primers were used: upstream, 5’-CCGCTCGAGATACTCTAAGGAGACATCAA-3’, and downstream, 5’-ATAAGAATGCGGCCGCTGCATTTGATTGGCTTTATT-3’. The gray background of the primer sequences represented protective bases, and the underlined parts were the enzyme cleavage sites for Xho I (upstream) and Not I (downstream). These enzyme cleavage sites facilitate the subsequent connection of the target gene with the psi-checkII vector. PCR amplification was carried out according to the instructions provided with the kit (Accurate, cat. AG12301, Changsha, China). In accordance with the instructions provided with the pGM-T vector ligation kit (Tiangen, cat. VT302-02, Beijing, China), the PCR products were ligated into the pGM-T vector with ligase. Then, the plasmids were transformed into competent TOP10 cells. After clone screening and amplification, the plasmids were extracted and sequenced. The sequencing results were aligned to a reference sequence (ENSBTAT0000028685.5) by using online software (https://www.novopro.cn/tools/muscle.html, accessed on 21 October 2023), and SNPs were analyzed with reference to the variant table in Ensembl (Ensembl genome browser 113, accessed on 11 October 2023). The samples were grouped according to *CSN3* 3’UTR polymorphism.

### 2.5. Quantitative Real-Time PCR (qPCR) of CSN1S1, CSN1S2, CSN2, and CSN3

To reduce the effect of the lactation stage on the expression of milk protein, 12 cows in the mid-lactation stage (weeks 10–35 of lactation) were selected from each group for qPCR. The primer sequences for *GAPDH*, *CSN1S1*, *CSN1S2*, *CSN2*, and *CSN3* are shown in Supplementary Table S1. The qPCR was performed according to the instructions provided with SuperReal PreMix Plus (SYBR Green). The expression of genes was normalized to that of *GAPDH*. The relative expression of each gene was calculated by the 2^−ΔΔCt^ method. The relative expression level of each gene was measured in triplicate, and the average value was taken for statistical analysis.

### 2.6. Western Blot Analysis of CSN3

Western blotting was used to assess CSN3 protein expression in milk somatic cell samples as described by Li [17]. Briefly, milk somatic cells obtained from fresh milk by centrifugation were lysed with a RIPA buffer (C500005-0100, Sangon Biotech, China). Then, equal amounts of protein were resolved via 12% SDS‒PAGE and transferred to 0.45 µm of NC membranes (YA1711, Solarbio, Beijing, China). After the membranes were blocked with a Protein-Free Rapid Blocking Buffer (PS108P, Epizyme, Shanghai, China) for 10 min, they were incubated with primary antibodies against CSN3 (1:5000, abx130922, Abbexa, Cambridge, UK) and GAPDH (1:2000, AF2819, Beyotime, Shanghai, China) at 4 °C overnight. Next, the membranes were washed three times with a TBST buffer and incubated separately with secondary antibodies at a dilution of 1:5000 [horseradish peroxidase (HRP)-labeled anti-rabbit IgG goat antibody (D110058, Sangon Biotech, Shanghai, China) and anti-mouse IgG goat antibody (D110087, Sangon Biotech, Shanghai, China)] for 2 h at 25 °C. After the membranes were washed twice to remove excess secondary antibodies, the protein bands were visualized using a highly sensitive enhanced chemiluminescence (ECL) reagent (C500044-0025, Sangon Biotech, Shanghai, China) and analyzed by ImageJ.

### 2.7. Bioinformatics Analysis of CSN3 3’UTR Polymorphisms 

The secondary structure and the minimum free energy (MFE) of the *CSN3* 3’UTR were predicted by RNAhybrid (BiBiServ2—RNAhybrid (uni-bielefeld.de)). The microRNAs that might target the bovine *CSN3* 3’UTR were predicted by TargetScan (TargetScanHuman 8.0, accessed on Release 8.0: September 2021), miRanda (http://www.microrna.org/, accessed on 6 September 2016), RNAhybrid 2.2 (BiBiServ2 - RNAhybrid, accessed on 18 September 2017), and miRmap (miRmap, accessed on 1 March 2022). The miRNAs with low expression levels in bovine mammary glands were deleted on the basis of miRNA expression profiles published in our previous study [17].

### 2.8. Dual Fluorescence Assay for miRNA Binding 

The recombinant T vector containing the *CSN3* 3’UTR sequence (Haplotype 1 and Haplotype 10) and the psi-check II vector were digested with two enzymes (*Xho I* and *Not I*, respectively). The target gene (*CSN3* 3’UTR) was ligated to a linear psi-check II vector at a molar ratio of 3:1 by using T4 ligase. By transforming, culturing, picking monoclones, and sequencing, the recombinant vectors psi-checkII-*CSN3* 3’UTR-1 (Haplotype 1) and psi-checkII-*CSN3* 3’UTR-2 (Haplotype 10) were constructed. MAC-T cells and 293T cells were seeded in 24-well plates at 1 × 10^5^ with DMEM (HyClone, Cat. No. SH30022.01, Shanghai, China) and 10% fetal bovine serum (Every Green, Cat. No. 11011-8611). After 24 h, the cells were cotransfected with the psi-check II recombinant vector and miRNA. The transfection doses were as follows: 800 ng/well for the psi-checkII recombinant plasmid, 30 pmol/well for the miRNA mimics or miRNA NC, and 1 μL for Lipofectamine^TM^ 3000 (Invitrogen, L3000008). After 48 h of transfection, a Dual Luciferase Reporter Gene Assay Kit (Beyotime Company, Cat. No. RG027) was used to detect Renilla fluorescence and firefly fluorescence. The relative fluorescence value (Renilla fluorescence value/firefly fluorescence value) was calculated.

Based on the dual fluorescence results of the above two psi-check II recombinant vectors, only bta-miR-708 significantly decreased the dual fluorescence ratio of psi-checkII-*CSN3* 3’UTR-2. According to the bta-miR-708 binding site prediction, there were two possible binding sites in the *CSN3* 3’UTR (Haplotype 10). To verify whether bta-miR-708 could directly bind to the psi-checkII-*CSN3* 3’UTR-2, three pairs of primers were designed (Supplementary Table S2). Mutations at miRNA binding sites were introduced during primer design. Binding site 1 of bta-miR-708 was mutated to construct the recombinant vector M1. Binding site 2 of bta-miR-708 was mutated to construct the recombinant vector M2. The two binding sites of bta-miR-708 were simultaneously mutated to construct the recombinant vector M3. The psi-checkII-*CSN3* 3’ UTR-2 was used as a template to amplify the mutated vector (M1, M2 and M3) by reverse amplification. The PCR mixture included 2 μL of upstream primer (10 μM), 2 μL of downstream primer (10 μM), 2 μL of plasmid template, 25 μL of 2x*ApexHF* FS PCR Master Mix (Accurate, cat. AG12202, China), and 19 μL of RNase-free ddH_2_O. The amplification conditions were predenaturation at 94 °C for 30 s, denaturation at 98 °C for 10 s, annealing at 55 °C for 15 s, and elongation at 72 °C for 2 min for a total of 30 cycles, followed by elongation at 72 °C for 2 min. The original recombinant vector (psi-checkII-*CSN3* 3’UTR-2) was digested with *Dpn*I. The nucleotide sequences of all the recombinant vectors were confirmed by sequencing. Using the original psi-checkII-*CSN3* 3’UTR-2 as the control, bta-miR-708 was cotransfected into cells with every recombinant vector, and dual fluorescence values were measured as described above.

### 2.9. Assessment of Dairy Herd Improvement (DHI) Data

The regular DHI samples were analyzed by Henan Dairy Herd Improvement Co., Ltd. (Zhengzhou, China) using a Danish Foss Milk Analyzer (FOSS, Hilleroed, Denmark). DHI data, including 305-d milk production, fat-corrected milk, fat (%), protein (%), and fat/protein ratio, were analyzed. The analysis was performed according to the *CSN3* 3’UTR polymorphism grouping.

### 2.10. Genomic DNA Sequencing of the CSN3 3’UTR

Genomic DNA sequences corresponding to the *CSN3* 3’UTR were verified by sequencing. DNA was extracted from the milk somatic cell samples according to the instructions provided with the TIANamp Genomic DNA Kit (DP304-02, Tiangen, China). The above upstream and downstream primers for the *CSN3* 3’ UTR were used to amplify the genomic DNA samples. Due to the insertion of an 1864 bp intron, the corresponding genomic DNA fragment of the *CSN3* 3’ UTR should be 2071 bp. The PCR mixture included 2 μL of DNA template, 2 μL of upstream primer (10 μM), 2 μL of downstream primer (10 μM), 25 μL of ApexHF HS DNA Polymerase FS Master Mix (Accurate, AG12202, Changsha, China), and 19 μL of RNase-free H_2_O. The amplification conditions were predenaturation at 94 °C for 1 min, denaturation at 98 °C for 5 s, annealing at 55 °C for 5 s, and elongation at 72 °C for 20 s for a total of 35 cycles, followed by elongation at 72 °C for 5 min. The amplified fragments of the correct length were used for sequencing after being ligated to the pGM-T vector as mentioned above. The sequencing results were aligned with reference sequences (ENSBTAT00000028685.5) in the Ensemble database.

### 2.11. Statistical Analysis

Comparisons between the two groups were analyzed by the unpaired *t* test in SPSS version 22. Comparisons between multiple groups were performed using a one-way ANOVA in SPSS version 22. The results are shown as means ± standard errors. A *p* value less than 0.05 was considered to indicate statistical significance.

## 3. Results

### 3.1. CSN3 3’UTR Polymorphisms

The full-length *CSN3* 3’UTR was successfully amplified from 50 samples from Chinese Holstein cows (Supplementary Figure S1). Sequencing and alignment of the *CSN3* 3’UTR revealed 18 mutation sites and 16 haplotypes in the 207 bp long *CSN3* 3’UTR (Table 1). The 50 samples were classified by different haplotypes, as shown in Table 1. Compared with the reference sequence (ENSBTAT0000028685.5), 16 samples belonged to Haplotype 1 (0 mutations) and 17 samples belonged to Haplotype 10 (7 mutations), which accounted for the majority of the 50 samples. The other haplotypes in the samples showed little change on the basis of zero mutations or seven mutations, and the number of samples was small. The seven mutant bases of Haplotype 10 all had SNP IDs, and the mutation frequency of each mutant base was more than 50%. The follow-up data were divided into two groups according to the two main haplotypes, which were named the Haplotype 1 group and Haplotype 10 group.

### 3.2. The Effect of CSN3 3’UTR Polymorphisms on the Gene Expression of CSN1S1, CSN1S2, CSN2, and CSN3 

A total of 12 dairy cows at the mid-lactation stage were selected from the Haplotype 1 group and the Haplotype 10 group for qPCR detection and analysis. As shown in Table 2, there was no significant difference in the relative gene expression of three casein genes (*CSN1S1*, *CSN1S2*, and *CSN2*) between the two groups, but there was a significant difference between the groups for CSN3 expression. The gene expression of *CSN3* in the Haplotype 1 group was significantly greater than that in the Haplotype 10 group.

### 3.3. The Effect of CSN3 3’UTR Polymorphisms on the CSN3 Protein

**Figure 1:**
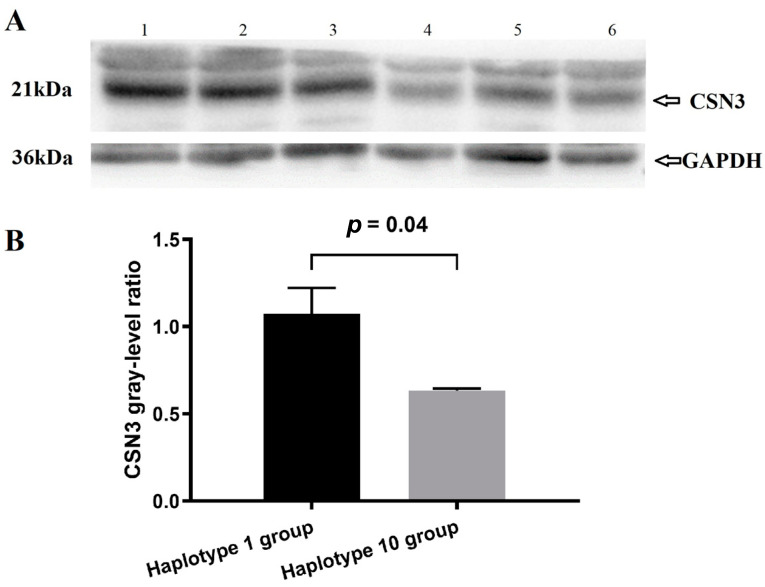
The effect of CSN3 3’UTR polymorphisms on CSN3 protein expression. (**A**) CSN3 protein expression was assessed by Western blotting. Samples 1-3 were from the Haplotype 1 group (0-mutations), and samples 4-6 were from the Haplotype 10 group (7-mutations). (**B**) Grayscale analysis of CSN3 protein expression. CSN3 gray-level ratio = CSN3 grayscale value/GAPDH grayscale value. The statistical differences between two groups were analyzed using a *t*-test.

Analysis of the CSN3 gray-level ratio revealed that the expression level of κ-casein in the Haplotype 1 group was significantly greater than that in the Haplotype 10 group (Figure 1A,B). The results for CSN3 protein expression were consistent with those for *CSN3* gene expression.

### 3.4. The Effect of CSN3 3’UTR Polymorphisms on Its Secondary Structure and Possibly Binding miRNAs 

The secondary structure and the minimum free energy of the two major haplotypes in the *CSN3* 3’UTR (Haplotype 1 and Haplotype 10) are shown in Table S3. Four online websites (TargetScan, miRanda, RNAhybrid 2.2, and miRmap) were used to predict which miRNAs might bind to the *CSN3* 3’UTR; the results are shown in Table S4. After removing miRNAs with low expression in bovine mammary glands, bta-miR-193a-3p, bta-miR-193b, bta-miR-2284w, bta-miR-2285f, bta-miR-2904, and bta-miR-708 were screened for a subsequent dual fluorescence assay.

### 3.5. The Effect of CSN3 3’UTR Polymorphisms on miRNA Binding 

**Figure 2:**
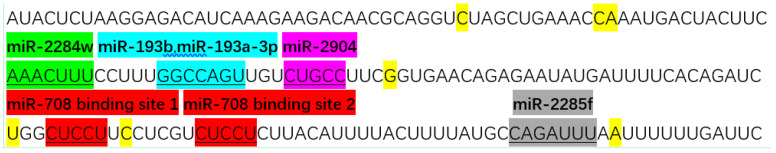
The possible binding sites between the seed sequences of the six miRNAs and the CSN3 3’ UTR. Different colors correspond to different miRNA binding sites. The reference sequence is obtained from NCBI (NM_174294.2) and belongs to the Haplotype 10 group (7-mutations). Bases with a bright yellow background are mutated bases. The underlined base is the corresponding binding site in the seed region of miRNA.

**Figure 3:**
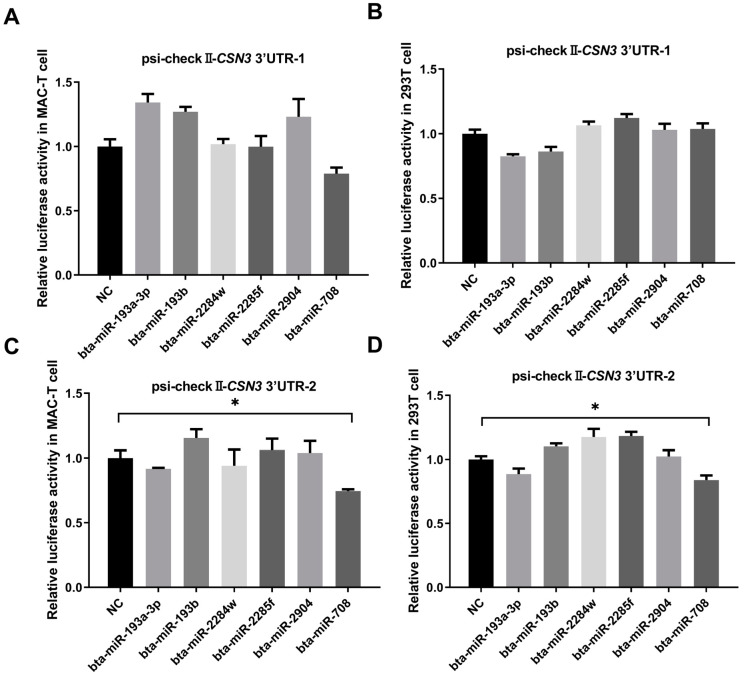
The dual-fluorescence assay results for six miRNAs and two recombinant vectors containing the psi-checkII-CSN3 3’ UTR (Haplotype 1 and Haplotype 10). Psi-checkII-*CSN3* 3’ UTR-1 was the recombinant vector with Haplotype 1. Psi-checkII-*CSN3* 3’ UTR-2 was the recombinant vector with Haplotype 10. The left panel shows the results for MAC-T cells, and the right panel shows the results for 293T cells. (**A**) represents the co-transfection of Psi-checkII-CSN3 3’UTR-1 and six miRNAs into MAC-T cells. (**B**) represents the co-transfection of Psi-checkII-CSN3 3’UTR-1 and six miRNAs into 293T cells. (**C**) represents the co-transfection of Psi-checkII-CSN3 3’UTR-2 and six miRNAs into MAC-T cells. (**D**) represents the co-transfection of Psi-checkII-CSN3 3’UTR-2 and six miRNAs into 293T cells. * indicates a significant difference between the two groups, i.e., *p* < 0.05.

**Figure 4:**
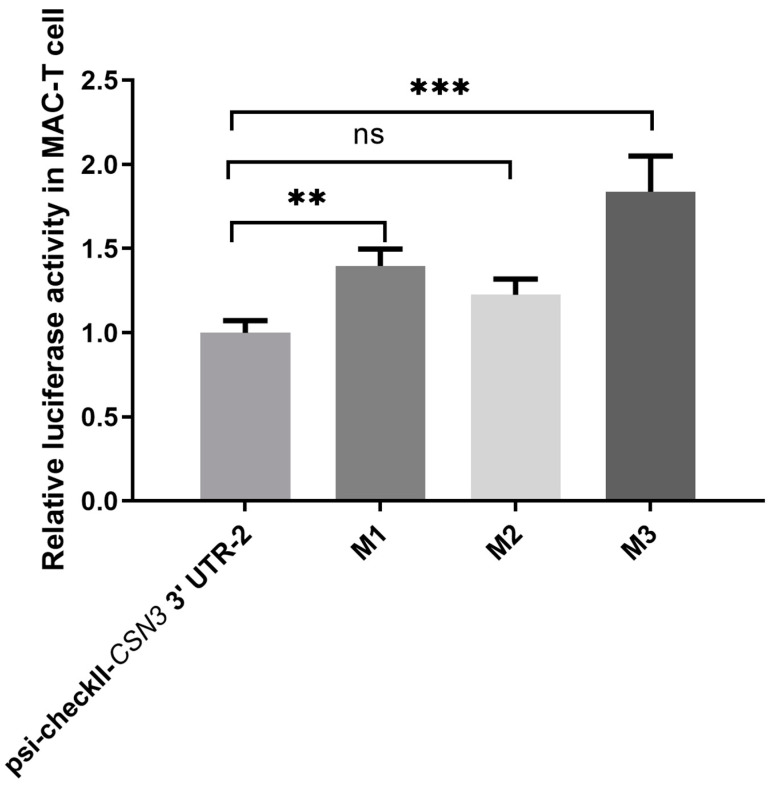
The dual-fluorescence analysis results for bta-miR-708 and three mutated recombinant vectors. M1 is the recombinant vector for mutated potential binding site 1. M2 is the recombinant vector for mutated potential binding site 2. M3 is the recombinant vector for mutated potential binding site 1 and mutated potential binding site 2. ** indicates a significant difference between the two groups, i.e., *p* < 0.01. *** indicates a significant difference between the two groups, i.e., *p* < 0.001. ns indicates no significant difference between the two groups.

The possible sites for the binding of the six miRNAs to the *CSN3* 3’UTR are shown in Figure 2. The dual-fluorescence analysis results are shown in Figure 3. In both MAC-T cells and 293T cells, the dual-fluorescence ratio of psi-checkII-*CSN3* 3’UTR-1 was not significantly decreased by the six miRNAs (*p* > 0.05) (Figure 3A,B). These results suggest that none of the six miRNAs targeted the *CSN3* 3’UTR with Haplotype 1 (0 mutations). However, bta-miR-708 significantly decreased the dual fluorescence of psi-checkII-*CSN3* 3’ UTR-2 in both MAC-T cells and 293T cells (*p* < 0.05) (Figure 3C,D), which indicates that bta-miR-708 could bind to the *CSN3* 3’ UTR with Haplotype 10 (7 mutations). According to our predictions, bta-miR-708 has two possible binding sites (Figure 2). The dual-fluorescence results for three mutated recombinant vectors (M1, M2 and M3) compared with psi-checkII-*CSN3* 3’ UTR-2 are shown in Figure 4; the findings indicate that binding site 1 more strongly bound to bta-miR-708 than binding site 2.

### 3.6. The Effect of CSN3 3’UTR Polymorphisms on DHI Data in Dairy Cows

The DHI raw data of 50 Chinese Holstein cows for March and August 2022 are shown in Supplementary Tables S5 and S6. On the basis of the *CSN3* 3’ UTR sequencing results for the aforementioned 50 cattle, the samples were divided into two groups: the Haplotype 1 group and the Haplotype 10 group: the Haplotype 1 group from March 2022 included cattle No. 4, 6, 8, 14, 16, 21, 22, 25, 28, 29, 32, 36, 38, 40, 42, and 43; the Haplotype 10 group included cattle No. 3, 7, 9, 10, 11, 15, 18, 19, 24, 26, 31, 34, 39, 41, 44, 45, and 47. To test the stability of the results, the DHI data from August 2022 were also analyzed. Over time, the number of milking cows decreased beginning in March 2022. The Haplotype 1 group included cattle No. 4, 8, 25, 28, 29, 32, and 36 in August 2022, and the Haplotype 10 group included cattle No. 10, 11, 19, 26, and 31. There were no significant differences between the two groups in 305-d milk production, fat-corrected milk, fat (%), protein (%), or the fat/protein ratio (see Table 3 and Table 4). Because this grouping was based on the 3’UTR polymorphisms of κ-casein and because κ-casein is a milk protein, we performed a statistical analysis of the milk protein content over a six-month period from March to August 2022. As shown in Table 5, there was no significant difference in the milk protein content between the two groups.

### 3.7. The Corresponding Genomic DNA Sequence of the CSN3 3’UTR

Three samples from the Haplotype 1 group and three samples from the Haplotype 10 group were selected for genomic DNA sequencing of the *CSN3* 3’UTR (results showed in Figure S2). Therefore, the mutated bases were derived from bovine genomic DNA. Moreover, the genomic DNA sequences of the three samples from the Haplotype 1 group were identical. The genomic DNA sequences of the three samples from the Haplotype 10 group were also identical. This suggests that no additional mutations occurred at the genomic DNA level, according to the grouping method of *CSN3* 3’UTR polymorphisms. In addition, no mutated bases were found in the 1864-bp intron region in the six tested samples.

## 4. Discussion

Because of the difficulty in obtaining ruminant mammary gland samples, it is challenging to study gene or protein expression in the mammary gland. Killing cows by electroshock or obtaining needle biopsies by microsurgery are methods for obtaining precise bovine mammary gland samples, but both methods are invasive [17,18]. Milk somatic cells, which can be obtained noninvasively, are an alternative sample option to study gene expression in the mammary gland [19,20,21,22]. In this study, cows with somatic cell counts less than 500,000 per milliliter were analyzed.

In the past, the study of gene polymorphisms was carried out at the genomic DNA level. This study explored *CSN3* polymorphisms at the mRNA level, which was useful for narrowing the scope of this study. The full length of the *CSN3* 3’UTR is 207 bp at the mRNA level and 2071 bp at the corresponding genomic DNA level because of the insertion of an 1864 bp intron. Surprisingly, pGM-T vector linkage sequencing revealed that there were two main types of *CSN3* 3’UTR polymorphisms: Haplotype 1 (mutation-free) and Haplotype 10 (simultaneous mutation at seven sites). This is the first time that such a regular mutation has been found, and the seven mutation sites all have SNP IDs.

The gene expression of four caseins (*CSN1S1*, *CSN2*, *CSN1S2,* and *CSN3*) was examined, and it was found that the gene expression of *CSN3* significantly differed between the two groups according to *CSN3* 3’UTR polymorphism grouping. This may be due to the variation of miRNA binding caused by the *CSN3* 3’ UTR polymorphism, resulting in the difference of *CSN3* gene expression levels. Western blot analysis revealed that the expression of the CSN3 protein in the Haplotype 1 group was greater than that in the Haplotype 10 group—a finding that was consistent with the *CSN3* gene expression results. The assessment of the κ-casein content in milk may be a more intuitive indicator. However, at present, the purity of the bovine κ-casein standard obtained from Sigma‒Aldrich (CAS No. 9000-71-9) is only ≥70%; therefore, the test results may be unreliable. An ELISA kit (CAS No. RD-CSN3-B, Reddot Biotech, Canada) for measuring the κ-casein content was also used in this study, but the OD values of some samples were lower than the minimum limit of detection without dilution of the milk; therefore, there were no results for some samples. Further exploration is needed to detect κ-casein in milk. DHI data are used as standard measurements to evaluate the milk production performance of dairy cows [23]. DHI measurements include milk yield, fat (%), protein (%), dry matter, urea nitrogen, and somatic cell number [24]. In this study, cows were divided into two groups by *CSN3* 3’UTR polymorphisms. However, there were no significant differences in DHI parameters, including 305-d milk production, fat-corrected milk, fat (%), protein (%), or fat/protein ratio, between the two groups. Since these are quantitative traits, it is possible that increasing the number of samples will increase the difference between the two groups, which needs to be further confirmed. The small number of samples (n = 50) used for the DHI analysis may affect the significance of the statistical results, which is a limitation of this analysis.

The 3’-UTR follows the coding region and is the terminal region of the transcript. The 3’UTR of a gene is usually a region regulated by miRNAs [25]. miRNAs are noncoding RNAs that regulate gene expression at the posttranscriptional level and usually bind to the 3’UTR of target genes, causing translational repression or degradation of their target mRNAs [26]. There are no reports on miRNA regulation of the bovine *CSN3* 3’UTR. Some prediction-level miRNA-targeting casein studies have been performed. For example, it was predicted that T>A (rs109787476) on 6:85656841 in the bovine *CSN3* 3’UTR would result in the loss of the bta-miR-496 binding site and gain of the bta-miR-2284w binding site and that C>A (ss7626433382) on 6:85658779 in the bovine *CSN3* 3’UTR would result in the loss of the bta-miR-193a-3p binding site [27]. However, based on high-throughput sequencing results, the expression level of bta-miR-496 was very low in the mammary glands at different lactation stages [17]; therefore, it was not used in this study. Six miRNAs (bta-miR-193a-3p, bta-miR-193b, bta-miR-2284w, bta-miR-2285f, bta-miR-2904, and bta-miR-708) were selected for subsequent analyses. According to the dual-fluorescence analysis, only bta-miR-708 significantly downregulated the expression of the psi-checkII-*CSN3* 3’UTR-2 but did not affect the expression of the psi-checkII-*CSN3* 3’UTR-1. Then, we further verified that bta-miR-708 could directly bind to the psi-checkII-*CSN3* 3’UTR-2 by mutating the predicted binding site on the *CSN3* 3’UTR. Therefore, *CSN3* polymorphisms indeed caused a change in the binding capacity of bta-miR-708. This may be one reason why the gene and protein expression of *CSN3* with Haplotype 10 were significantly lower than those of *CSN3* with Haplotype 1. The predicted seed sequence binding region of bta-miR-708 on the *CSN3* 3’UTR did not have mutations, and the mutations occurred near the seed binding region. This was similar to the results of Zhang et al., who found that a mutation (rs1044129 A>G) located 13 bp upstream of this binding site affected miR-367 binding to RYR3 3’UTR [28]. All this suggests that mutations in the vicinity of miRNA seed sequence binding sites also affected miRNA targeted binding.

## 5. Conclusions

This study was the first to describe the characteristics of *CSN3* 3’UTR polymorphisms in Chinese Holstein cows and revealed that they had a significant effect on the gene and protein expression of κ-casein. The potential mechanism of action might involve the difference in miRNA binding ability caused by *CSN3* 3’UTR polymorphisms. At present, there are no differences in milk production data. Because *CSN3* 3’UTR mutations are related to the expression level of *CSN3* in the mammary gland, it is necessary to screen cows with the detected *CSN3* 3’UTR polymorphisms during breeding. The milk from cows with Haplotype 1 is more advantageous for future dairy processing and production.

## Figures and Tables

**Table 1:** The mutation site, mutation frequency, and the haplotype of CSN3 3’UTR in 50 cattle.

	Allele Position in 3’UTR and SNP ID	26 A>G rs450438851	27 C>T (No SNP ID)	36 T>C (No SNP ID)	37 T>C rs209024052	48 T>C rs209359743	49 G>A rs135528518	80 T>C (No SNP ID)	92 A>G rs135796226	104 A>T (No SNP ID)	122 C>T rs134221650	129 T>C (No SNP ID)	131 T>C rs136843602	141 T>C (No SNP ID)	154 T>C (No SNP ID)	170 G>A rs134516686	178 A>G (No SNP ID)	183 T>C (No SNP ID)	187 T>C (No SNP ID)
Haplotype/ Number of Mutant Bases (Sample Number)																			
**Haplotype 1**/No mutation point (No. 4,6,8,14,16,21,22, 25,28,29,32,36,38,40,42,43)																			
Haplotype 2/One mutation point (No. 5)																			C
Haplotype 3/One mutation point (No. 12)			T																
Haplotype 4/One mutation point (No. 48)										T									
Haplotype 5/Two mutation points (No. 2)											T							C	
Haplotype 6/Two mutation points (No. 35)													C			A			
Haplotype 7/Four mutation points (No. 50)											T	C	C			A			
Haplotype 8/Six mutation points (No. 13,46)					C	C	A		G		T		C						
Haplotype 9/Six mutation points (No. 1,17)					C	C	A		G				C			A			
**Haplotype 10**/Seven mutation points (No. 3,7,9,10,11,15,18, 19,24,26,31,34,39,41,44,45, 47)					C	C	A		G		T		C			A			
Haplotype 11/Seven mutation points (No. 33,37)				C		C	A		G		T		C			A			
Haplotype 12/Eight mutation points (No. 20)					C	C	A		G		T		C		C	A			
Haplotype 13/Eight mutation points (No. 23)					C	C	A		G		T		C			A	G		
Haplotype 14/Eight mutation points (No. 27)					C	C	A	C	G		T		C			A			
Haplotype 15/Eight mutation points (No. 49)					C	C	A		G		T		C			A			C
Haplotype 16/Nine mutation points (No. 30)		G			C	C	A		G		T		C	C		A			
Base mutation frequency		2%	2%	2%	52%	56%	56%	2%	56%	2%	56%	2%	60%	2%	2%	56%	2%	2%	4%

Note: Use ENSBTAT0000028685.5 as the reference sequence. The numbers in parentheses represent the sample numbers of 50 cattle, and samples with consistent sequencing results are merged into one line. Two haplotypes (Haplotype 1 and Haplotype 10) with larger sample sizes are bold. The gray background highlights the bases where the mutation frequency is higher than 50%.

**Table 2:** The relative quantitative analysis of the four casein genes in the Haplotype 1 group and the Haplotype 10 group.

	Gene	*CSN1S1*	*CSN1S2*	*CSN2*	*CSN3*
Group					
Haplotype 1 group (n = 12)		1 ± 0.41	1 ± 0.41	1 ± 0.30	1 ± 0.24
Haplotype 10 group (n = 12)		0.49 ± 0.22	0.78 ± 0.32	1.40 ± 0.81	0.04 ± 0.02
*p*		0.339	0.341	0.622	0.001

**Table 3:** Difference analysis of milk production performance between the Haplotype 1 group and the Haplotype 10 group. (DHI data from March 2022).

	Index	305-d Milk Production	Fat-Corrected Milk	Fat (%)	Protein (%)	Fat/Protein Ratio
Group						
Haplotype 1 group (n = 16)		7899.93 ± 456.12	29.81 ± 3.08	3.51 ± 0.15	3.41 ± 0.09	0.95 ± 0.03
Haplotype 10 group (n = 17)		7825.38 ± 662.29	31.58 ± 2.86	3.56 ± 0.17	3.58 ± 0.10	0.96 ± 0.04
*p*		0.928	0.678	0.828	0.241	0.822

**Table 4:** Difference analysis of milk production performance between the Haplotype 1 group and the Haplotype 10 group. (DHI data from August 2022).

	Index	305-d Milk Production	Fat-Corrected Milk	Fat (%)	Protein (%)	Fat/Protein Ratio
Group						
Haplotype 1 (n = 7)		9057.62 ± 303.89	33.08 ± 3.30	3.18 ± 0.39	3.54 ± 0.10	0.90 ± 0.10
Haplotype 10 (n = 5)		9071.40 ± 1278.61	27.89 ± 5.35	3.96 ± 0.38	3.86 ± 0.24	1.04 ± 0.10
*p*		0.990	0.404	0.197	0.207	0.360

**Table 5:** Difference analysis of milk protein (%) between the Haplotype 1 group and the Haplotype 10 group. (Data from March 2022 to August 2022).

	Month	March	April	May	June	July	August
Group							
Haplotype 1		3.41 ± 0.09	3.17 ± 0.06	3.23 ± 0.07	3.52 ± 0.09	3.50 ± 0.10	3.54 ± 0.10
Haplotype 10		3.58 ± 0.10	3.36 ± 0.11	3.33 ± 0.11	3.67 ± 0.14	3.61 ± 0.16	3.86 ± 0.24
*p*		0.241	0.117	0.459	0.362	0.576	0.207

Note: the number of samples from the two groups in each month above will decrease with the decrease of milking cows.

## Data Availability

The original contributions presented in the study are included in the article and Supplementary Materials. Further inquiries can be directed to the corresponding authors.

## Supplementary Materials

The following supporting information can be downloaded at: https://www.mdpi.com/article/10.3390/ani14233462/s1, Figure S1. The electrophoresis bands for 50 amplified fragments of the full-length *CSN3* 3’UTR. Figure S2. The alignment results for the genomic DNA sequences corresponding to two types of *CSN3* 3’UTR polymorphic sequences (Haplotype 1 and Haplotype 10) with reference sequences. Table S1. The primers for the four casein genes used for qPCR. Table S2. The primers used for the mutagenesis of bta-miR-708 binding sites. Table S3. The sequence and secondary structure predictions for the two types of *CSN3* 3’UTR polymorphisms (Haplotype 1 and Haplotype 10). Table S4. The miRNAs potentially targeting the bovine *CSN3* 3’UTR. Tables S5 and S6. The DHI data for March and August 2022.

## References

[1] Inoue R., Tsukahara T. (2021). Composition and physiological functions of the porcine colostrum. Anim. Sci. J..

[2] Paul A., Martin F., Simard B., Scher J., Gaiani C., le Floch-Fouere C., Jeantet R., Burgain J. (2023). Deciphering the segregation of proteins in high-protein dairy powders after spray-drying. J. Dairy. Sci..

[3] Fan X., Zhang Z., Qiu L., Zhang Y., Miao Y. (2019). Polymorphisms of the kappa casein (CSN3) gene and inference of its variants in water buffalo (*Bubalus bubalis*). Arch. Anim. Breed..

[4] Madende M., Osthoff G. (2019). Comparative genomics of casein genes. J. Dairy. Res..

[5] Day L., Williams R.P., Otter D., Augustin M.A. (2015). Casein polymorphism heterogeneity influences casein micelle size in milk of individual cows. J. Dairy Sci..

[6] Dadousis C., Biffani S., Cipolat-Gotet C., Nicolazzi E.L., Rosa G.J.M., Gianola D., Rossoni A., Santus E., Bittante G., Cecchinato A. (2017). Genome-wide association study for cheese yield and curd nutrient recovery in dairy cows. J. Dairy. Sci..

[7] Dadousis C., Biffani S., Cipolat-Gotet C., Nicolazzi E., Rossoni A., Santus E., Bittante G., Cecchinato A. (2016). Genome-wide association of coagulation properties, curd firmness modeling, protein percentage, and acidity in milk from Brown Swiss cows. J. Dairy Sci..

[8] Hobor S., Kunej T., Dovc P. (2008). Polymorphisms in the kappa casein (CSN3) gene in horse and comparative analysis of its promoter and coding region. Anim. Genet..

[9] Caroli A.M., Chessa S., Erhardt G.J. (2009). Invited review: Milk protein polymorphisms in cattle: Effect on animal breeding and human nutrition. J. Dairy Sci..

[10] Djedovic R., Bogdanovic V., Perisic P., Stanojevic D., Popovic J., Brka M. (2015). Relationship between genetic polymorphism of κ-casein and quantitative milk yield traits in cattle breeds and crossbreds in Serbia. Genetika.

[11] Ibeagha-Awemu E.M., Kgwatalala P., Zhao X. (2008). A critical analysis of production-associated DNA polymorphisms in the genes of cattle, goat, sheep, and pig. Mamm. Genome.

[12] Mohr U., Koczan D., Linder D., Hobom G., Erhardt G. (1994). A single point mutation results in A allele-specific exon skipping in the bovine αS1-casein mRNA. Gene.

[13] Rando A., Di Gregorio P., Ramunno L., Mariani P., Fiorella A., Senese C., Marletta D., Masina P. (1998). Characterization of the CSN1A^G^ allele of the bovine αs1-casein locus by the insertion of a relict of a long interspersed element. J. Dairy Sci..

[14] Prinzenberg E.M., Weimann C., Brandt H., Bennewitz J., Kalm E., Schwerin M., Erhardt G. (2003). Polymorphism of the bovine CSN1S1 promoter: Linkage mapping, intragenic haplotypes, and effect on milk production traits. J. Dairy Sci..

[15] Damiani G., Caroli A., Leone P., Budelli E., Florio S. (2001). Effect of Bov-A2 Sine elements on quantitative traits. Proceedings of the XIV National Congress ASPA.

[16] Cosenza G., Mauriello R., Garro G., Auzino B., Iannaccone M., Costanzo A., Chianese L., Pauciullo A. (2019). Casein composition and differential translational efficiency of casein transcripts in donkey’s milk. J. Dairy Res..

[17] Li W.Q., Li W.L., Wang X.Y., Zhang H.L., Wang L.F., Gao T.Y. (2022). Comparison of miRNA profiles in milk-derived extracellular vesicles and bovine mammary glands. Int. Dairy J..

[18] Cui X., Hou Y., Yang S., Xie Y., Zhang S., Zhang Y., Zhang Q., Lu X., Liu G., Sun D. (2014). Transcriptional profiling of mammary gland in Holstein cows with extremely different milk protein and fat percentage using RNA sequencing. BMC Genom..

[19] Krappmann K., Weikard R., Kühn C. (2012). Evaluation of a replacement method for mammary gland biopsies by comparing gene expression in udder tissue and mammary epithelial cells isolated from milk. Res. Vet. Sci..

[20] Cánovas A., Rincón G., Bevilacqua C., Islas-Trejo A., Brenaut P., Hovey R.C., Boutinaud M., Morgenthaler C., VanKlompenberg M.K., Martin P. (2014). Comparison of five different RNA sources to examine the lactating bovine mammary gland transcriptome using RNA-Sequencing. Sci. Rep..

[21] Suárez-Vega A., Gutiérrez-Gil B., Klopp C., Robert-Granie C., Tosser-Klopp G., Arranz J.J. (2015). Characterization and Comparative Analysis of the Milk Transcriptome in Two Dairy Sheep Breeds using RNA Sequencing. Sci. Rep..

[22] Michailidou S., Gelasakis A., Banos G., Arsenos G., Argiriou A. (2021). Comparative Transcriptome Analysis of Milk Somatic Cells During Lactation Between Two Intensively Reared Dairy Sheep Breeds. Front. Genet..

[23] Shock D.A., LeBlanc S.J., Leslie K.E., Hand K., Godkin M.A., Coe J.B., Kelton D.F. (2015). Exploring the Characteristics and Dynamics of Ontario Dairy Herds Experiencing Increases in Bulk Milk Somatic Cell Count During the Summer. J. Dairy Sci..

[24] Haltia L., Honkanen-Buzalski T., Spiridonova I., Olkonen A., Myllys V. (2006). A study of bovine mastitis, milking procedures and management practices on 25 Estonian dairy herds. Acta Vet. Scand..

[25] Xu H., Shao J., Fang J., Yin B., Zhang L., Zhang J., Xia G. (2021). miR-381 Targets KCTD15 to Regulate Bovine Preadipocyte Differentiation In Vitro. Horm. Metab. Res..

[26] Langschied F., Leisegang M.S., Brandes R.P., Ebersberger I. (2023). ncOrtho: Efficient and reliable identification of miRNA orthologs. Nucleic Acids Res..

[27] Vanvanhossou S.F.U., Giambra I.J., Yin T., Brügemann K., Dossa L.H., König S. (2021). First DNA Sequencing in Beninese Indigenous Cattle Breeds Captures New Milk Protein Variants. Genes.

[28] Zhang L., Liu Y., Song F., Zheng H., Hu L., Lu H., Liu P., Hao X., Zhang W., Chen K. (2011). Functional SNP in the microRNA-367 binding site in the 3’UTR of the calcium channel ryanodine receptor gene 3 (RYR3) affects breast cancer risk and calcification. Proc. Natl. Acad. Sci. USA.

