# Supplementary material for: Polymorphism of the CSN3 3’UTR in Dairy Cows Causes Changes in bta-miR-708 Binding Ability and κ-Casein Expression
Source: Animals (Basel). 2024 Nov 29;14(23):3462. doi: 10.3390/ani14233462 (PMC11640128; doi:10.3390/ani14233462)
Supplement: Supplementary file 1 [file animals-14-03462-s001.zip › animals-3281695-supplementary.pdf]

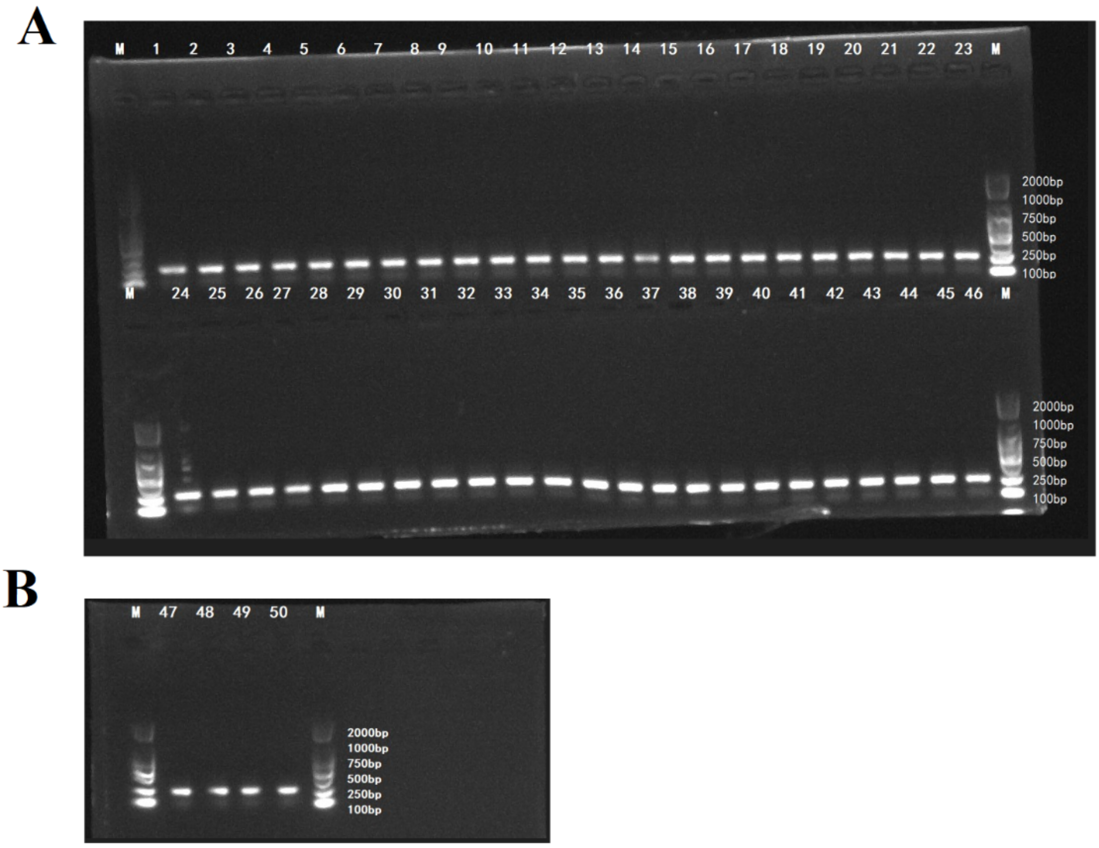

Figure S1. The electrophoresis bands for 50 amplified fragments of *CSN3* 3'UTR full-length.

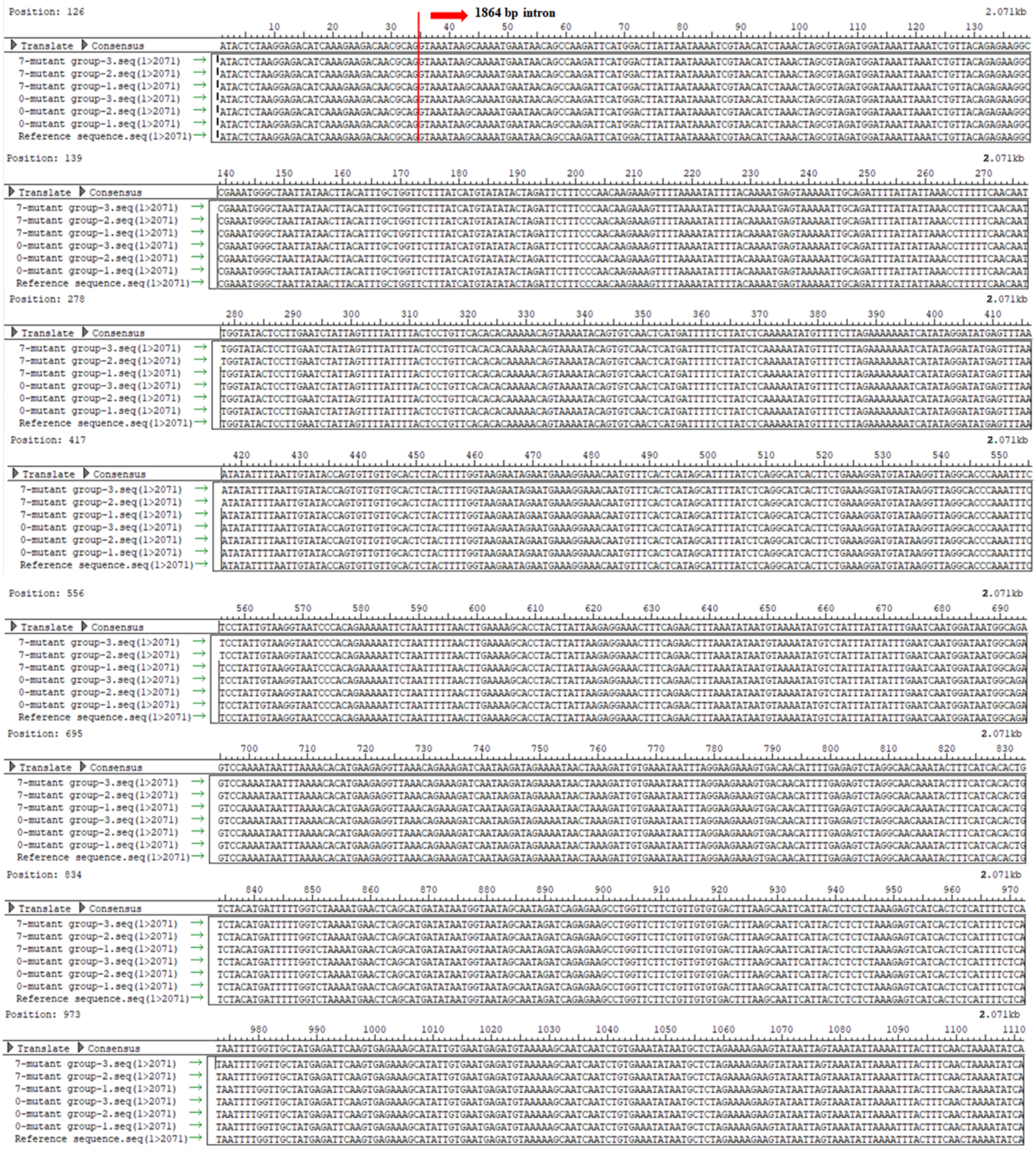
1864 bp intron

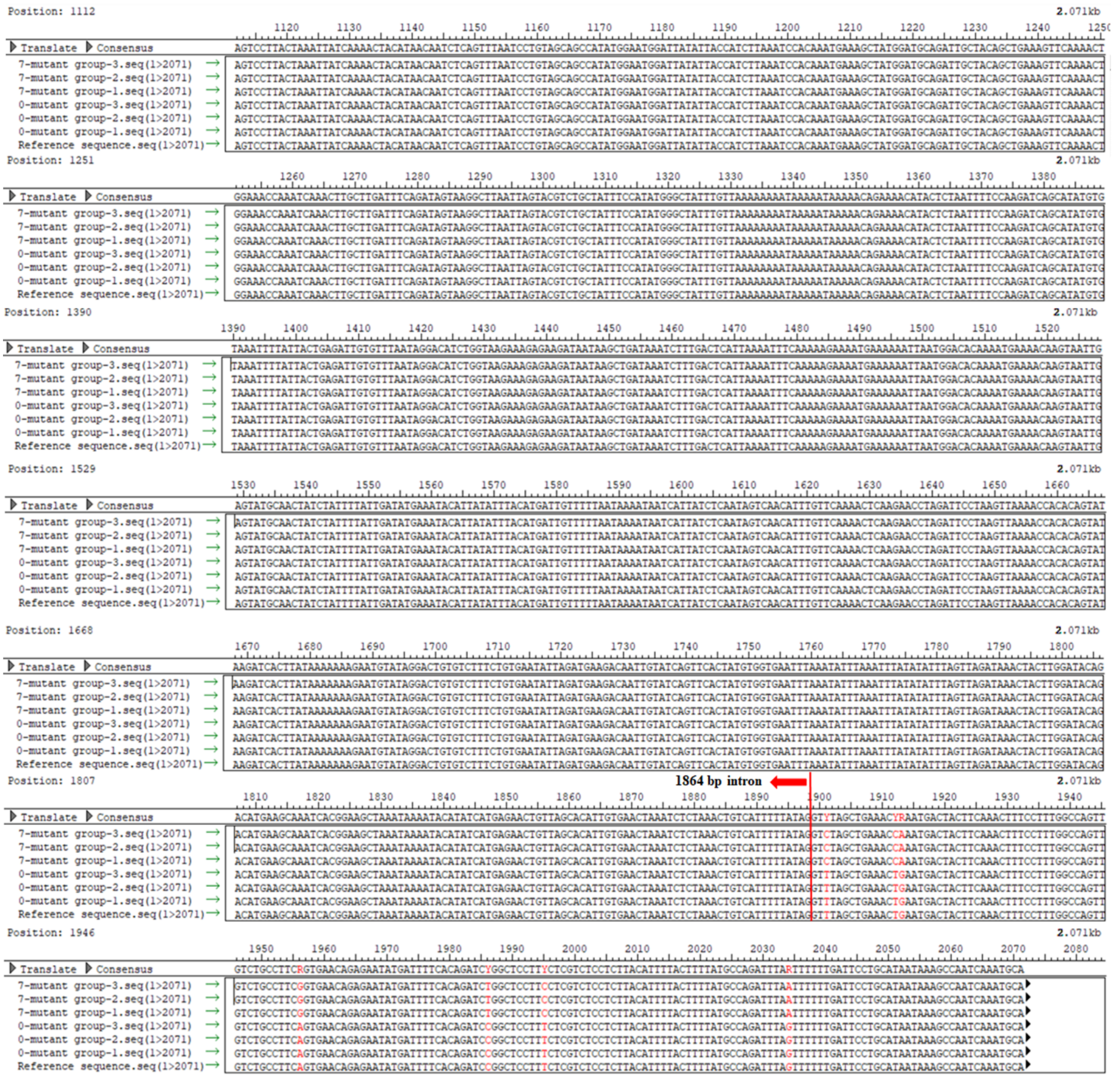

Figure S2. Alignment result of genomic DNA sequences corresponding to two types of *CSN3* 3'UTR polymorphic sequences (Haplotype 1 and Haplotype 10).

Table S1. Primer used for quantitative real-time PCR

Gene Symbol	Accession Number	Primer Sequence	Size(bp)
CSN1S1	BC109618.1	5’-TACCTGTCTTGTGGCTGTTGC	239
		5’- CCTTTTGAATGTGCTTCTGCTC	
CSN1S2	BC114773.1	5’-GCCTGGACTACTTGTCTTCCTTTTA	243
		5’- TCCTCTTCATTTGCGTTCCTTAC	
CSN2	BC111172.1	5’-AGTGAGGAACAGCAGCAAACAG	317
		5’- AGCAGAGGCAGAGGAAGGTG	
CSN3	BC102120.1	5’- TTCAACTGCGGTCTAAATACTCTAAG	194
		5’- TCAAAAAACTAAATCTGGCATAAAAG	
GAPDH	NM_001034034.2	5’- AGCGAGATCCCTGCCAACATCAAG	221
		5’- GCAGGAGGCATTGCTGACAATCT	

Table S2. Primer design of recombinant vectors for mutagenesis of bta-miR-708 possible binding sites

Recombinant vector name	Mutation site	Primer sequences
M1	Binding site 1	F:5'-TTCACAGATCTGGATAATTCCTCGTCTC CTCTTACATTTTACTTTTATGC<br>R:5'-GAGGAGACGAGGAATTATCCAGATCTG TGAAAATCATATTCTCTGTTCA
M2	Binding site 2	F:5'-GCTCCTTCCTCGTATAATCTTACATTTT ACTTTTATGCCAGATTTAATTT<br>R:5'-AGTAAAATGTAAGATTATACGAGGAAG GAGCCAGATCTGTGAAAATCATA
M3	Two binding sites	F:5'-GATAATTCCTCGTATAATCTTACATTTT ACTTTTATGCCAGATTTAATTT<br>R:5'-AGTAAAATGTAAGATTATACGAGGAATTAT CCAGATCTGTGAAAATCATA

Note: The underlined part of the sequence is the binding site, and the gray background is the mutated base.

Table S3. The sequence and the secondary structure prediction for the two types of *CSN3* 3'UTR polymorphism (Haplotype 1 and Haplotype 10).

Haplotype	The number of SNPs	Sample number	The RNA sequence of the 3' UTR	The predicted secondary structure and the minimum free energy (MFE)
1	0	4,6,8,14,16,21,22,25,28,<br>29,32,36,38,40,42,43	AUACUCUAAGGAGACAUCAAAGAAGA<br>CAACGCAGGUUUAGCUGAAACUGAAU<br>GACUACUUCAAACUUUCCUUUGGCCA<br>GUUGUCUGCCUUCAGUGAACAGAGAA<br>UAUGAUUUUCACAGAUCCGGCUCCUU<br>UCUCGUCUCCUCUUACAUUUUACUUU<br>UAUGCCAGAUUUAGUUUUUUGAUUCC<br>UGCAUAAUAAAGCCAAUCAAAUGCA	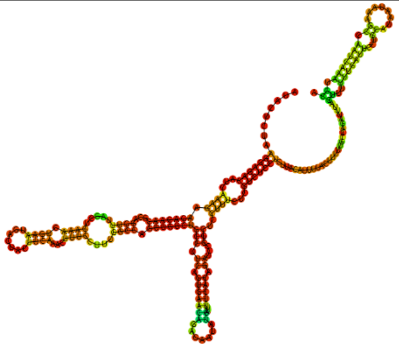<br>MFE=-37.70 kcal/mol
10	7	3,7,9,10,11,15,18,19,24,<br>26,31,34,39,41,44,45,47	AUACUCUAAGGAGACAUCAAAGAAGA<br>CAACGCAGGUCUAGCUGAAACCAAAU<br>GACUACUUCAAACUUUCCUUUGGCCA<br>GUUGUCUGCCUUCGGUGAACAGAGAA<br>UAUGAUUUUCACAGAUCUGGCUCCUU<br>CCUCGUCUCCUCUUACAUUUUACUUU<br>UAUGCCAGAUUUAAUUUUUUGAUUC<br>CUGCAUAAUAAAGCCAAUCAAAUGCA	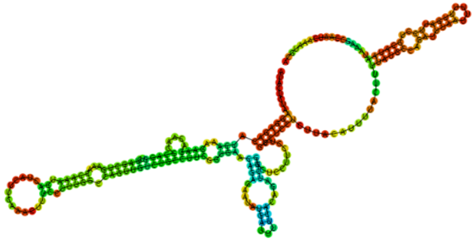<br>MFE=-39.70 kcal/mol

Table S4. The miRNAs potentially targeting the bovine *CSN3* 3 'UTR

miRNA name and mature miRNA sequence	TargetScan	miRanda	RNAhybrid 2.2(http://bibiserv.techfak.uni-bielefeld.de/rnahybrid)	miRmap (miRmap web (ezlab.org))	Expression level
>bta-miR-193a-3p<br>AACUGGCCUACA<br>AAGUCCCAGU	Hit	Hit<br>-16.87<br>kCal/Mol	MFE: -22.4 kcal/mol (no SNP)<br>position 45<br>target 5' A AAU ACUUCAAACUUUCC U G 3'<br>ACUG GACU UU GGCCAGUU<br>UGAC CUGA AA CCGGUCAA<br>miRNA 3' C CAU 5'<br>MFE: -21.8 kcal/mol (7 SNPs)<br>position 56<br>target 5' U UCAA CCUUU G 3'<br>ACU ACUUU GGCCAGUU<br>UGA UGAAA CCGGUCAA<br>miRNA 3' CCC CAU 5'	Hit	middle
>bta-miR-193b<br>AACUGGCCCACA<br>AAGUCCCGCUUU	Hit	Hit<br>-16.17<br>kCal/Mol	MFE: -20.9 kcal/mol (no SNP)<br>position 63<br>target 5' A CCUUU G 3'<br>ACUUU GGCCAGUU<br>UGAAA CCGGUCAA<br>miRNA 3' UUUCGCCC CAC 5'<br>MFE: -20.9 kcal/mol (7 SNPs)<br>position 63<br>target 5' A CCUUU G 3'<br>ACUUU GGCCAGUU<br>UGAAA CCGGUCAA<br>miRNA 3' UUUCGCCC CAC 5'	Hit	middle
> bta-miR-496<br>UGAGUAUUACA<br>UGGCCAAUCUC	No	No	MFE: -20.2 kcal/mol (no SNP)<br>position 71<br>target 5' U UG U 3'<br>UUGGCCA GU UC<br>AACCGGU UA AG<br>miRNA 3' CUCU ACAU UG U 5',<br>MFE: -20.2 kcal/mol (7 SNPs)	No	low

			position 71<br>target 5' U UG U 3'<br>UUGGCCA GU UC<br>AACCGGU UA AG<br>miRNA 3' CUCU ACAU UG U 5'		
> bta-miR-2284w<br>AAGAGUUUGUU<br>CGGGUUUCUC	No	Hit<br>-5.7<br>kCal/Mol	MFE: -17.1 kcal/mol (no SNP)<br>position 42<br>target 5' U GAA CUACUU C 3'<br>GAAACU UGA CAAACUUU<br>CUUUGG GCU GUUUGAGA<br>miRNA 3' CU U A 5'<br>MFE: -18.5 kcal/mol (7 SNPs)<br>position 42<br>target 5' U AAAU CUACUU C 3'<br>GAAACC GA CAAACUUU<br>CUUUGG CU GUUUGAGA<br>miRNA 3' CU G U A 5'	No	middle
>bta-miR-2285ab<br>AAAACCUGAAU<br>GAACUUCUUGG	No	Hit<br>-16.17<br>kCal/Mol	MFE: -20.3 kcal/mol (no SNP)<br>position 19<br>target 5' A ACAACG A 3'<br>AAGAAG CAGGUUU<br>UUCUUC GUCCAAA<br>miRNA 3' GG AAGUAA A 5'<br>MFE: -17.9 kcal/mol (7 SNPs)<br>position 19<br>target 5' A ACAACG C 3'<br>AAGAAG CAGGU<br>UUCUUC GUCCA<br>miRNA 3' GG AAGUAA AAA 5'	No	low
>bta-miR-2285f<br>AAAACCUGAAU<br>GAACUUUUUGG	No	Hit<br>-13.47<br>kCal/Mol	MFE: -17.8 kcal/mol (no SNP)<br>*Position: 16*<br>target 5' A AGA ACG A 3'<br>UCAAAGA CA CAGGUUU<br>GGUUUUU GU GUCCAAA<br>miRNA 3' CAA AA A 5'	Hit	middle

			MFE: -15.4 kcal/mol (7 SNPs)<br><br>*Position: 16*<br>target 5' A AGA ACG C 3'<br>UCAAAGA CA CAGGU<br>GGUUUUU GU GUCCA<br>miRNA 3' CAA AA AAA 5'		
>bta-miR-2904<br>GGGAGCCUCG<br>GUUGGCCUC	No	No	MFE: -23.0 kcal/mol (no SNP)<br>*Position: 73*<br>target 5' U UU U G U 3'<br>GGCCAG G CU CC<br>CCGGUU C GA GG<br>miRNA 3' CU GG UCC G 5'<br>MFE: -23.0 kcal/mol (7 SNPs)<br>*Position: 73*<br>target 5' U UU U G U 3'<br>GGCCAG G CU CC<br>CCGGUU C GA GG<br>miRNA 3' CU GG UCC G 5'	Hit	middle
>bta-miR-2285g<br>AAACCUGAACA<br>AGCUUUUUGG	No	No	MFE: -18.5 kcal/mol (no SNP)<br>*Position: 19*<br>target 5' A ACAACG A 3'<br>AAGAAG CAGGUU<br>UUUUUC GUCCAA<br>miRNA 3' GG GAACAA 5'<br>MFE: -16.4 kcal/mol (7 SNPs)<br>*Position: 19*<br>target 5' A ACAACG C 3'<br>AAGAAG CAGGU<br>UUUUUC GUCCA<br>miRNA 3' GG GAACAA AA 5'	Hit	middle

>bta-miR-708<br>AAGGAGCUUACA<br>AUCUAGCUGGG	No	No	MFE: -20.4 kcal/mol (no SNP)<br>*Position: 119*<br>target 5’ A CCUUUCUCGU C 3’<br>UCCGGCU CUCCU<br>GGGUCGA GAGGA<br>miRNA 3’ UCUAACAUUC A 5’<br>MFE: -20.0 kcal/mol (7 SNPs)<br>*Position: 112*<br>target 5’ U CU C 3’<br>UCA C AGAU GGCUCCUU<br>GGU G UCUA UCGAGGAA<br>miRNA 3’ G C A ACAU 5’	Hit	middle
>bta-miR-28<br>AAGGAGCUCACA<br>GUCUAUUGAG	No	No	MFE: -19.8 kcal/mol (no SNP)<br>*Position: 112*<br>target 5’ U C CC U 3’<br>UCA AGAU GGCUCCUU<br>AGU UCUG UCGAGGAA<br>miRNA 3’ G UA ACAC 5’<br>MFE: -19.8 kcal/mol (7 SNPs)<br>*Position: 112*<br>target 5’ U C CU C 3’<br>UCA AGAU GGCUCCUU<br>AGU UCUG UCGAGGAA<br>miRNA 3’ G UA ACAC 5’	Hit	middle

Note: The predicted miRNAs were coming from four software’s analyses combined with high-throughput sequencing of bovine mammary glands (Wenqing Li, et al., 2022). MFE indicates the minimum free energy.

Table S5 DHI data for 50 cows in March 2022.

Serial number	The number of the cow	Milk yield	Fat (%)	Protein (%)	Somatic cell counts (10^4/mL)	Urea nitrogen	Days of lactation	Fat/Protein ratio	Fat Corrected milk	305-d milk
1	18030	38.4	2.44	3.43	8	15.4	113	0.71	26.5	7909
2	17010	38.6	3.13	3.1	4	18.4	178	1.16	32.5	7731
3	15024	34	3.86	3.17	1.22	20.5	152	1.07	21.9	6116
4	18046	30.4	3.77	3.67	6	20.3	130	1.00	27.8	7148
5	17026	33.2	3.18	3.1	3.7	18.4	178	1.16	36.9	10372
6	19050	27.6	3.86	3.82	2	18.7	159	0.99	42.8	6814
7	17032	34.6	3.31	4.27	6	22.4	179	0.79	27.3	7068
8	19006	44.2	2.14	3.17	16	20.5	152	1.22	32.9	9199
9	18027	17.4	4.93	2.96	2	20.3	122	0.96	36.6	13390
10	17030	36.2	2.63	3.82	8	17.2	113	0.70	14.1	4903
11	17029	27	4.21	3.88	16	16.9	147	0.80	15.9	5594
12	18006	34.8	3.36	3.13	1.07	4.2	115	0.79	19.7	6568
13	18035	38	3.08	4.15	3.2	17.9	138	1.02	11.6	2501
14	19003	28.8	2.58	3.27	18	16.2	177	0.89	26.3	9079
15	19038	39.6	3.37	3.6	11	24.9	424	0.88	56.0	11085
16	18036	38.4	2.61	3.63	17	18.4	110	0.99	31.6	10599
17	18042	27	3.39	4.02	10	20.6	66	1.05	50.7	7705
18	14026	32.8	3.6	3.33	2	17.9	138	0.79	28.7	5328
19	16007	51.8	2.84	2.96	0.96	20.3	112	0.71	26.5	7981

20	20008	38.3	2.43	4.23	11.5	19.1	82	1.12	13.3	7909
21	18032	37.2	4.03	3.13	4	17.4	114	1.07	65.4	8723
22	19001	41	2.85	2.9	3	14.8	37	1.41	21.8	8490
23	19027	31.8	2.89	3.82	21	18	92	0.88	43.6	8422
24	15017	29	3.36	4.45	3	22.4	358	1.11	33.0	6950
25	14057	44.2	3.8	3.82	2.1	18.7	159	0.93	35.3	7502
26	14044	36	3.1	3.48	4	20.6	183	1.16	41.9	9939
27	17019	29.6	2.92	3.54	1	14.6	75	0.87	25.2	7970
28	19040	35.4	3.03	3.51	17	18.7	111	0.74	26.5	8670
29	18007	31.6	4.09	3.17	3	18.6	122	0.86	15.2	5098
30	19025	32	3.19	3.83	4	17.2	153	0.89	25.3	5541
31	14055	32.8	3.66	3.47	18	18.2	169	1.09	33.8	9438
32	19037	36.6	2.91	3.53	3	17.2	109	0.81	29.7	8162
33	19029	30.6	3.33	3.32	2	18.7	105	0.92	34.5	7826
34	19039	26	3.52	3.64	3	17.3	220	0.97	23.4	6123
35	14026	32.8	3.6	3.38	15	18.3	262	0.89	31.7	6581
36	19036	36.2	2.79	3.41	15	17.6	161	0.96	24.1	6543
37	19054	22.6	3.38	3.71	19	22.2	269	1.11	26.0	6401
38	19022	24	3.29	4.05	5	19.1	170	0.96	16.8	5413
39	1324	32.6	3.36	3.09	2	19.5	262	1.03	41.5	9209
40	17016	11.8	4.22	3.03	5	21	163	0.95	29.9	9645
41	17027	16.6	3.59	3.32	2	18.7	105	1.00	25.6	5374
42	19023	17.6	4.1	3.1	6	19.4	153	0.90	32.3	7607
43	19047	22.2	3.79	3.2	2	18.5	70	0.91	24.1	6605
44	18021	22.6	3.35	3.7	18	18.8	176	0.91	42.1	7271

45	18003	14.6	4.72	3.64	3	17.3	220	0.97	31.8	6508
46	19021	25	3.01	3.1	2	19.3	128	0.98	30.3	6736
47	16036	21	2.67	4.07	14	20	150	0.93	23.4	7498
48	19024	14.8	3.89	3.82	2	17.6	161	1.01	30.4	7255
49	18039	20.4	2.74	3.35	18.4	22.1	183	1.01	25.3	6174
50	16041	19.4	3.12	3.88	19.6	18.1	147	0.99	26.2	6116

Table S6 DHI data for 23 cows in August 2022.

Note: Since some cows stopped producing milk in August, the DHI sample size was reduced to 23 cows. The blank cell of the table is unchecked.

Serial number	The number of the cow	Milk yield	Fat (%)	Protein (%)	Somatic cell counts (10^4/mL)	Urea nitrogen	Days of lactation	Fat/Protein ratio	Fat Corrected milk	305-d milk
1	18030	14.2	3.43	4.07	13	17.7	289	0.84	19.7634	9923.537
2	17010	16	4.6	4.14	13	16	303	1.11	24.8663	8995.451
4	18046	29	3.51	3.79	9.2	14.9	344	0.93	48.2672	10227.07
8	19006	18.4	2.01	3.8	9.8	15.1	353	0.53	26.3695	8289.413
10	17030	19.4	2.67	3.93	6	16.5	321	0.68	26.4081	8038.643
11	17029	17	4.87	4.26	13	15.8	607	1.14	43.3109	7704.736
12	18006	19	4.07	3.45	3.4	15.4	655	1.18	48.5842	8722.628
13	18035	23.8	4.38	3.96	2.4	18	251	1.11	34.1714	7948.3
19	16007	26.8	3.66	3.55	9.3	15.4	297	1.03	36.3922	14178.28
20	20008	20.8	3.01	3.47	5.6	16.5	241	0.87	26.2127	9533.898
25	14057	16	5.09	3.81	4	13.9	334	1.34	27.2339	8166.853
26	14044	14.6	4.1	3.11	15	13.2	242	1.32	18.9219	7625.587
27	17019	11	4.26	4.29	12	19.8	352	0.99	18.0115	9601.635

28	19040	18.8	3.48	3.35	1.4	13.9	303	1.04	28.8147	8350.997
29	18007	22.8	3.12	3.57	18.3	15.5	214	0.87	24.5503	9255.246
30	19025	9	5.09	3.71	21.6	17.5	5	1.37	7.5385	
31	14055	9.6	4.52	4.44	17.1	16.8	305	1.02	14.4196	7809.734
32	19037	28.8	2.96	3.27	1.4	14.4	246	0.91	36.4902	9769.524
33	19029	26	3.65	3.77	18.6	14.4	280	0.97	38.7682	6.1
36	19036	29	2.11	3.22	2.2	15.9	328	0.66	39.8244	9344.261
37	19054	5.6	4.85	4.14	20.7	17.1	369	1.17	11.0484	5828.926
42	19023	11	5.91	4.29	19.4	17.9	15	1.38	10.9966	
43	19047	19	4.04	3.77	6	12.3	325	1.07	32.2484	7561.307
